# An Intermediary Role of Adenine Nucleotides on Free Fatty Acids-Induced Hyperglycemia in Obese Mice

**DOI:** 10.3389/fendo.2019.00497

**Published:** 2019-08-06

**Authors:** Xiao Yang, Yang Zhao, Qi Sun, Yunxia Yang, Yan Gao, Wenhao Ge, Junhao Liu, Xi Xu, Jianfa Zhang

**Affiliations:** Center for Molecular Metabolism, Nanjing University of Science and Technology, Nanjing, China

**Keywords:** plasma 5′-AMP, type 2 diabetes, hyperglycemia, free fatty acids, nucleotides release

## Abstract

Increased plasma free fatty acids (FFA) level plays a central role in the development of type 2 diabetes. Our previous studies have shown that plasma 5′-adenosine monophosphate (5′-AMP) elevates and acts as a potential upstream regulator of hyperglycemia in diabetic *db*/*db* mice. The relationship between FFA and plasma adenosine nucleotides in type 2 diabetes remains unclear. Here we found that plasma 5′-AMP level was also increased in diabetic mice induced by a high-fat diet and streptozotocin (HFD-STZ), as observed in diabetic *db*/*db* mice. The metabolites of adenosine nucleotides in plasma were increased in obese mice compared to lean mice. An acute oil gavage to lean mice increased both FFA and plasma purine metabolites, accompanying with glucose intolerance. 5′-AMP administration resulted in an increase in dose-dependent purine metabolites and different levels of glucose intolerance. FFA induced a release of adenine nucleotides from cultural human umbilical vein endothelial cells (HUVECs) prior to induction of their apoptosis. FFA also reduced red blood cells (RBCs) resistance to reactive oxygen species (ROS), leading to hemolysis, thereby increasing plasma nucleotides. Our results suggest that plasma adenine nucleotides play an intermediary role in FFA-induced glucose intolerance and hyperglycemia in obese mice.

## Introduction

Obesity is generally associated with an increased risk of insulin resistance and type 2 diabetes (1). Lipolysis is elevated in obesity, leading to increased level of FFA in plasma (2). However, the enlarged adipose tissue is not the direct cause of insulin resistance. Transgenic mice with increased fatty acid re-esterification in adipose tissue show obesity without increasing serum FFA and insulin resistance (3). While transgenic mice named A-ZIP/F-1, without virtually white adipose tissue, have a high level of FFA, insulin resistance and hyperglycemia (4). Thus, FFA, rather than elevated fat mass, plays a central role in insulin resistance and the development of type 2 diabetes.

Several mechanisms have been proposed to explain how FFA inhibits insulin signaling. However, the molecular basis for increased FFA causing impaired insulin action and glucose metabolism in obesity remains unclear. Insulin resistance is characteristic of insulin insensitive and impaired glucose tolerance (5). Impaired glucose tolerance often presages the hyperglycemia (6). Adenine nucleotides play multiple roles in insulin resistance and diabetes. ATP can inhibit the stimulation effect of insulin on glycolysis in hepatocyte (7). Exogenous adenine nucleotides (ATP, ADP, and 5′-AMP) show stimulatory effects on gluconeogenesis in isolated hepatocyte (8). The infusion of adenine nucleotides into perfused rat livers results in the stimulation of hepatic glycogenolysis (9). Uric acid (UA), a metabolite of adenine nucleotides, has been shown to inhibit the phosphorylation of AMP kinase (AMPK) and stimulates gluconeogenesis in diabetes (10). These observations suggest that adenine nucleotides play a crucial role in regulating glucose metabolism.

In our previous studies, we have shown that plasma 5′-AMP elevates and plays an upstream role in hyperglycemia in obese *db/db* diabetic mice (11). An injection of 5′-AMP causes hyperglycemia, accompanying with the elevation of plasma 5′-AMP and its metabolite UA in wild type mice. Although cell injury induced by FFA increases extracellular level of 5′-AMP in cultural cells (11), it remains unclear whether plasma adenosine nucleotides regulate FFA-related hyperglycemia in type 2 diabetes. Here we showed that the plasma 5′-AMP or its metabolites increased in obese mice. Mice received 5′-AMP resulted in an increase in dose-dependent purine metabolites and different levels of glucose intolerance and hyperglycemia. We also showed two potential sources of increased plasma nucleotides in obese mice. Our results suggest that plasma adenine nucleotides play an intermediary role in FFA-induced glucose intolerance and hyperglycemia in obese mice.

## Materials and Methods

### Animals

Mice were obtained from SLACCAS (Shanghai Alac Laboratory Animal Co. Ltd., Shanghai, China). Male C57BL/6, C57BL/Ks *db/db*, C57BL/6 *ob/ob* mice and their homozygous lean littermates (+/+) were used in this study. Eight week-old mice were used except for mice fed on high fat diet (HFD). Animals were maintained under controlled environmental conditions of temperature (22 ± 2°C) and 12:12-h light-dark cycles, with the light on at 07:00 and off at 19:00, with free access to regular food and water. To create diet-induced insulin resistance animal models, 4-week-old male mice were randomly divided into two groups. Mice in HFD group were fed a high-fat-diet (60% of calories from fat, 20% from protein, and 20% from carbohydrates) for 10 weeks. To create diet-induced type 2 diabetes animal models, 4-week-old male mice were fed with HFD for 10 weeks and then injected intraperitoneally with streptozotocin (STZ) dissolved in 0.05 M citrate buffer (pH 4.5) at a dosage of 100 mg/kg body weight. Age matched control mice were injected with citrate buffer only. Mice were then kept on the same diet for the next 2 weeks. Diabetic mice were defined as having morning blood glucose levels >16.7 mmol/l. All procedures were approved by Animal Care and Use Committee at Nanjing University of Science and Technology.

### Treatment With Olive Oil, 5′-AMP and Glucose Tolerance Test

Eight-week old mice were fasted overnight and given 10 ml/kg of olive oil intragastrically at 07:00 on the following morning. Two hours after olive oil treatment, mice were applied to glucose tolerance test or sacrificed for the analysis of plasma FFA and nucleotides. For the glucose tolerance test, mice were injected intraperitoneally with glucose (2 g/kg body weight). 5′-AMP was solvated in saline and administered to mice by intraperitoneal injection in doses of 0.05 or 0.5 μmol/g body weight. One hour after the treatment of 5′-AMP, blood glucose, FFA and plasma nucleotides levels were measured. To evaluate the effect of 5′-AMP on glucose tolerance, mice were fasted overnight. 5′-AMP and glucose were injected at the same time. Whole blood was collected from the carotid arteries in anticoagulant tubes (EDTA). Blood samples were immediately centrifuged at 5,000 g for 5 min at 20°C. The plasma samples obtained were then stored on ice and immediately used. Blood glucose was determined using One Touch Ultra Blood Glucose Meter with 3 μl of the whole blood obtained by tail bleed.

### Measurement of Plasma FFA

The concentration of plasma FFA was measured using colorimetric assay kits (Jianchen Biotech, Inc., Nanjing, China).

### Analysis of Adenine Nucleotides and Metabolites With High Performance Liquid Chromatography (HPLC)

The plasma samples were deproteinized with ice-cold perchloric acid resulted in the final concentration of 0.4 N. After centrifugation at 12,000 g for 10 min (4°C), the supernatant was transferred to a clean tube and neutralized according to the procedure described previously (12). Samples were then analyzed using HPLC (Waters 1525 System; Millipore, Bedford, MA) equipped with Partisphere bonded phase C18 (reverse phase) column, according to the protocols described previously (13). Pure nucleotides and metabolites solutions were used to identify the peaks and obtain the calibration curves. Quantitation was based on the area under the peaks.

### Treating Human Umbilical Vein Endothelial Cells With FFA

HUVECs were cultured in M199 and DMEM with 20% fetal bovine serum (FBS), 100 U·100 μg^−1^·ml^−1^ penicillin- streptomycin, 2 mM L-glutamine, 5 g/ml endothelial cell growth supplement, and 17.85 IU/ml heparin at 37°C under 5% CO_2_-95% air. FFA stocks were oleic acid (OA), palmitic acid (PA) and mixture with a molar ratio of 2:1 and were prepared as described previously (14). Cells were cultured in 60 mm dishes until 80–90% confluence. Then 500 μL of PBS (10 mM sodium phosphate buffer pH7.4, 136 mM NaCl, 2.6 mM KCl) was used to replace the cultural medium, then indicated concentration of FFA was added. In some other cells (control groups), bovine serum albumin (BSA) without FFA was added. After 5 min, 400 μL of the extracellular solution were collected and deproteinized with 0.4 N perchloric acid. Nucleotides content was determined by HPLC. In some experiments, 100 μM glibenclamide was added into PBS 30 min prior to the addition of FFA. To evaluate FFA-induced apoptosis, HUVECs were treated with different concentrations of OA and PA for indicated time in culture medium. Cells were harvested by trypsin-EDTA treatment, washed with PBS and stained (10 min, at room temperature) with fluorescein-Annexin V and propidium iodide (PI) as instructed in manufacturer's protocol (Nanjing Jiancheng Bioengineering Institute, China). Labeled cells were analyzed by flow cytometry using a FACS Calibur flow cytometer.

### Treating RBCs and Plasma With H_2_O_2_

The separated RBCs from wild type mice were washed twice with five volumes of PBS containing 20 mM glucose. At last, one volume of PBS containing 20 mM glucose was added to RBCs. To determine the elimination rate of H_2_O_2_ and nucleotides releasing, plasma and washed RBCs were incubated with H_2_O_2_ or plus indicated concentration of FFA at 37°C for 10 min. Then the RBCs were centrifuged at 1,500 rpm for 10 min at 4°C. The supernates from RBCs and incubated plasma were used to determine the H_2_O_2_ content, nucleotides and hemolysis. H_2_O_2_ content was determined using xylenol orange method as described previously (15). Adenine nucleotides were separated and quantified by HPLC. Absorbance (540 nm) measured from supernatant was used for hemolysis analysis.

### Statistical Analysis

Data were presented as means ± S.D. Statistical analysis was performed with Student's *t*-test, one-way ANOVA with a Newman-Keuls *post hoc* test or two-way ANOVA. Significance was defined by *p* < 0.05.

## Results

### Plasma Adenine Nucleotides and the Metabolites Were Increased in Obese Diabetic Mice

**Figure 1:**
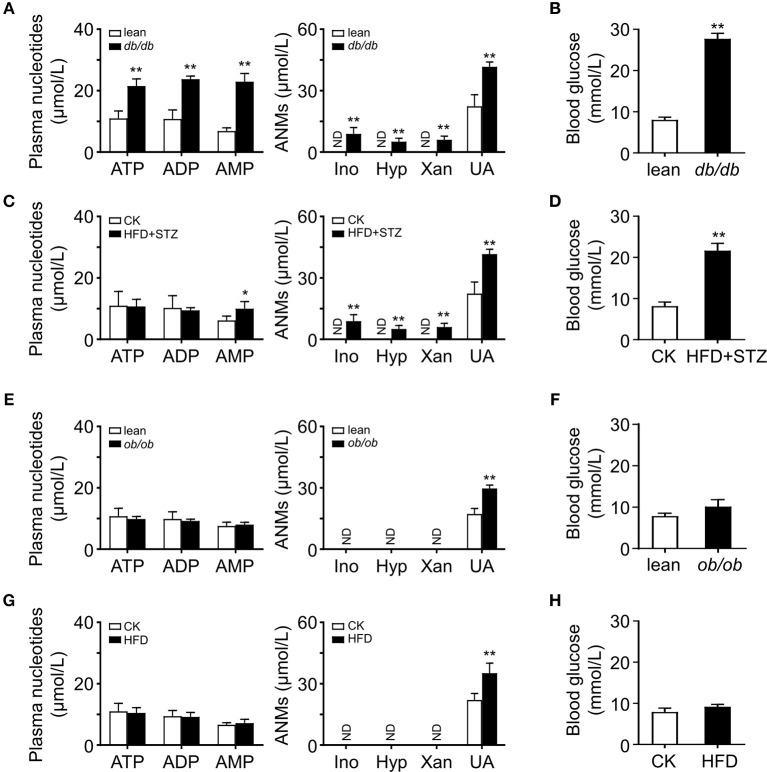
Activated adenine nucleotides metabolism in obese mice. HPLC analysis of plasma adenine nucleotides and its metabolites in *db/db* **(A)**, HFD-STZ **(C)**, *ob/ob* **(E)** and HFD induced **(G)** mice. Adenine nucleotides metabolites, Ino, Hyp, Xan, and UA were increased in the plasma of *db/db* and HFD-STZ mice while *ob/ob* and HFD mice only had increased level of plasma UA. *db/db* **(B)** and HFD+STZ **(D)** mice showed severe hyperglycemia while *ob/ob* **(F)** and HFD obese mice had normal blood glucose level **(H)**. ND, not detected. Data was expressed mean ± SD, with *n* = 5–6 in each group; *p < 0.05, **p < 0.01 compared with the control group (Student's *t*002Dtest).

The plasma 5′-AMP is elevated and results in hyperglycemia in obese diabetic *db/db* mice (11). To clarify whether this change of plasma purine nucleotides also occurs in other obese mice, we examined plasma levels of adenine nucleotide metabolites (ANMs) in several common obese mouse models. HPLC analysis showed that the plasma nucleotides and their metabolites, including inosine (Ino), hypoxanthine (Hyp), xanthine (Xan) and UA, increased in obese *db/db* mice and HFD-STZ mice, which had severe hyperglycemia symptoms (Figures 1A–D). However, in *ob/ob* mice and HFD-induced obese mice, we did not observe the increase of plasma adenine nucleotides; only found that their metabolite UA was increased (Figures 1E,G). Compared with *db/db* and HFD-STZ mice, *ob/ob* mice and HFD mice had no hyperglycemia symptoms (Figures 1F,H).

### Acute Oil Gavage Led to an Increase in Plasma ANMs

**Figure 2:**
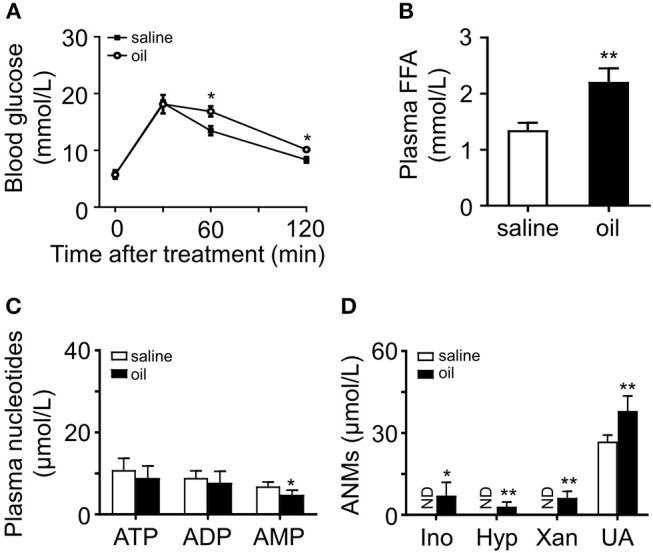
Effects of olive oil on glucose tolerance and plasma purine metabolism. **(A)** Olive oil induced glucose intolerance of wild type mice. **(B)** Plasma FFA increased significantly after oil administration. **(C,D)** Quantitation of adenine nucleotides and ANMs, including Ino, Hyp, Xan, and UA in the plasma of olive oil treated mice compared with those of the saline group. In olive oil treated mice, ANMs were elevated in the plasma while adenine nucleotides remain unchanged. ND, not detected. Data was expressed mean ± SD, with *n* = 5–8 in each group; analyses were performed using two-way ANOVA for glucose tolerance test and Student's *t*-test for FFA measurement and the quantitation of adenine nucleotides and ANMs; *p < 0.05, **p < 0.01 compared with the control group.

To investigate whether acute oil gavage induce changes in plasma purine nucleotide metabolites in mice, olive oil (10 ml/kg) was administered intragastrically into lean mice. Oil administration significantly increases plasma FFA level (16) and acute insulin resistance (17). In anticipation, the mice developed glucose intolerance 2 h after olive oil administration (Figure 2A), and plasma FFA levels increased significantly (Figure 2B). Then we analyzed adenine nucleotides and metabolites in plasma by HPLC. There was no significant change in plasma adenine nucleotide levels in mice after intragastric administration of olive oil (Figure 2C), but plasma ANMs increased significantly (Figure 2D).

### 5′-AMP Injection Caused Dose-Dependent Glucose Intolerance

**Figure 3:**
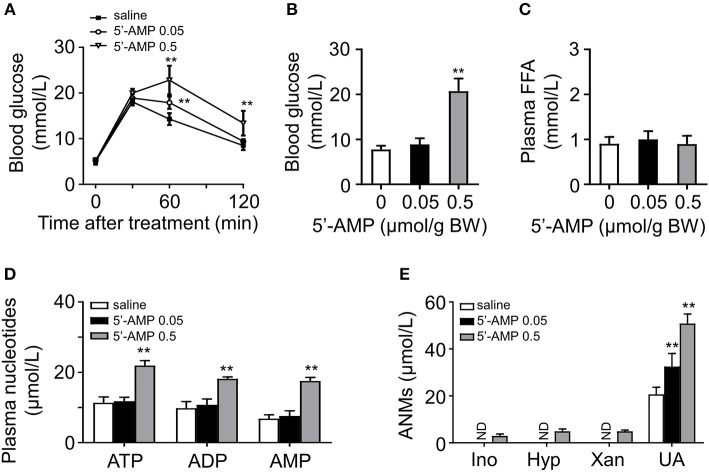
5′-AMP induced glucose intolerance and increased plasma ANMs level. **(A)** 5′-AMP (0.05 and 0.5 μmol/g body weight) induced glucose intolerance in wild mice. **(B)** Low-dose treatment with 5′-AMP did not influence blood glucose. **(C)** Plasma FFA level was not altered by 5′-AMP. **(D,E)** Quantitation of adenine nucleotides and ANMs in the plasma of mice treated with different doses of 5′-AMP. ND, not detected. Data was expressed mean ± SD, with *n* = 5–8 in each group; analyses were performed using two-way ANOVA for glucose tolerance test and Student's *t*-test for blood glucose, plasma FFA and quantitation of adenine nucleotides and ANMs; **p < 0.01 compared with the control group.

We have shown that administration of a high dose of 5′-AMP causes severe insulin resistance and hyperglycemia in mice (11). To elucidate the relationship between adenine nucleotides, FFA, and glucose intolerance, we injected different doses of 5′-AMP into lean mice. We found that mice received 5′-AMP exhibited dose-dependent glucose intolerance (Figure 3A). While low-dose 5′-AMP had no influence on blood glucose, high-dose 5′-AMP caused hyperglycemia in mice (Figure 3B). 5′-AMP treatment did not influence plasma FFA level even at a high dose (Figure 3C). Different doses of 5′-AMP have different effects on plasma adenine nucleotide and metabolite levels. As observed in *ob/ob* and HFD mice, lean mice received low dose of 5′-AMP only elevated plasma UA. High-dose of 5′-AMP caused to increase plasma ATP, ADP, 5′-AMP, and ANMs (Figures 3D,E).

### FFA Induced Apoptosis of Cultural HUVECs

**Figure 4:**
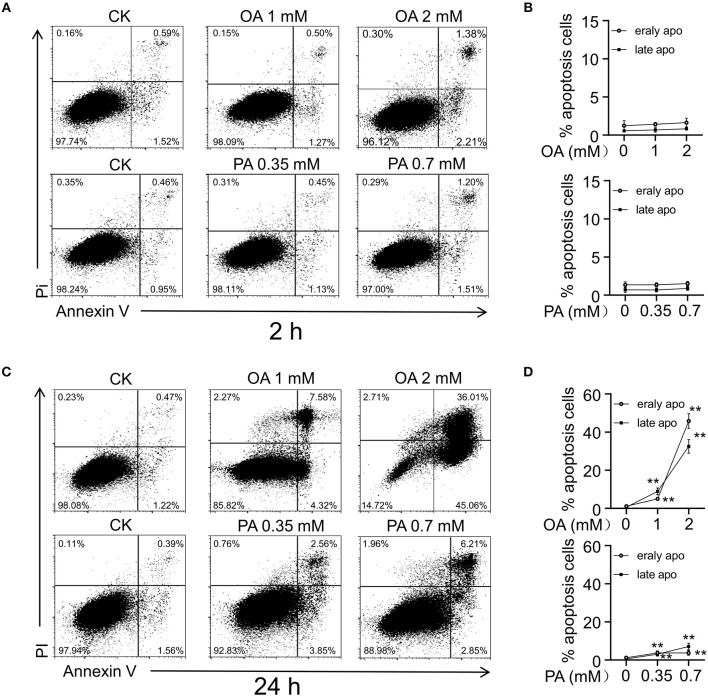
FFA induced cell injury in HUVECs. Flow cytometry analysis **(A,C)** and quantitation **(B,D)** of apoptosis in HUVECs induced by OA and PA. After 24 h of incubation, early apoptosis (Annexin-V), and late apoptosis (PI) induced by OA and PA increased in a dose dependent manner. However, early apoptosis and late apoptosis did not increase significantly in first 2 h. Typical results of three independent experiments were plotted. All data were expressed mean ± S.D, *n* = 3 in each group, **p < 0.01 compared to control group (one-way ANOVA with a Newman-Keuls *post hoc* test).

After 24- and 48-h treatment, FFA induced HUVECs injury in a dose- and time-dependent manner, resulting in nucleotides release into extracellular space (11). Then we examined the effects of OA (monounsaturated fatty acid) and PA (saturated fatty acid) on HUVECs apoptosis, respectively. After the treatment for 2 and 24 h, the cells were detached with trypsin and stained with fluorescein-Annexin V and propidium iodide (PI). OA and PA did not induce significant apoptosis at the first 2 h (Figures 4A,B). In accordance with previous study, both the early apoptosis and late apoptosis increased after 24-h treatment with OA or PA (Figures 4C,D).

### OA Induced Adenine Nucleotide Release Prior to Apoptosis of HUVECs

**Figure 5:**
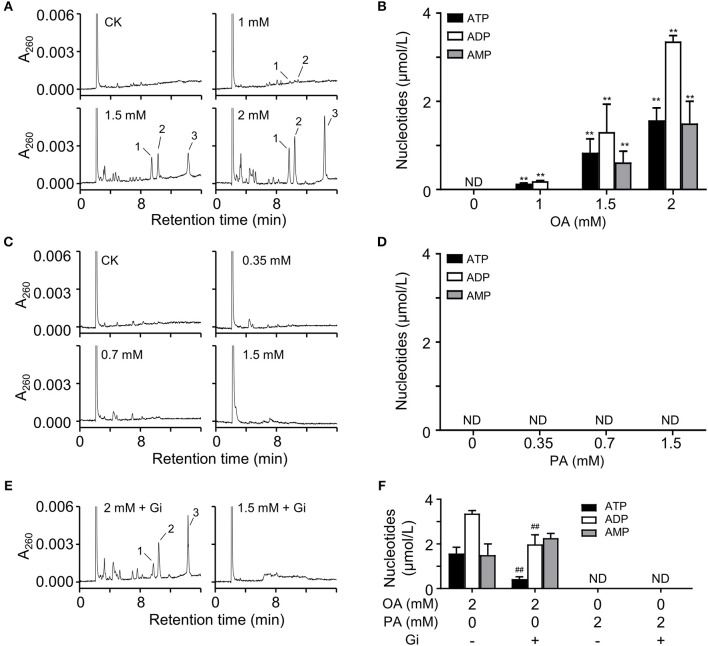
OA induced nucleotides releasing from HUVECs. **(A,C)** Representative HPLC profiles of nucleotides from the extracts of extracellular buffers in HUVECs incubated with various concentrations of OA and PA for 5 min. **(B,D)** Quantitation of ATP, ADP, and 5′-AMP from OA and PA treatment. **(E,F)** In the presence of 100 μM glibenclamide (Gi), OA-induced nucleotides release was partly inhibited. Peak 1: ATP; peak 2: ADP, peak 3: 5′-AMP. Typical results of three independent experiments were plotted. ND, not detected. All data were expressed mean ± S.D, *n* = 3 in each group; **p < 0.01 compared to control group (one-way ANOVA); ^##^*p* < 0.01 compared to OA or PA treated group (Student's *t*-test).

To investigate the effect of short-term FFA on HUVECs, we analyzed the extracellular nucleotides levels 5 min after FFA administration. We observed that OA induced the release of adenine nucleotides from HUVECs in a dose-dependent manner (Figure 5A). However, PA, even at relatively high concentration, failed to induce the release of adenine nucleotides from these cells (Figure 5C). Following quantitative analysis confirmed the observations (Figures 5B,D). Moreover, OA-induced nucleotides release was partly inhibited by glibenclamide, an inhibitor of the ATP-sensitive potassium channels (Figures 5E,F).

### H_2_O_2_ Induced the Release of Adenine Nucleotides From RBCs

**Figure 6:**
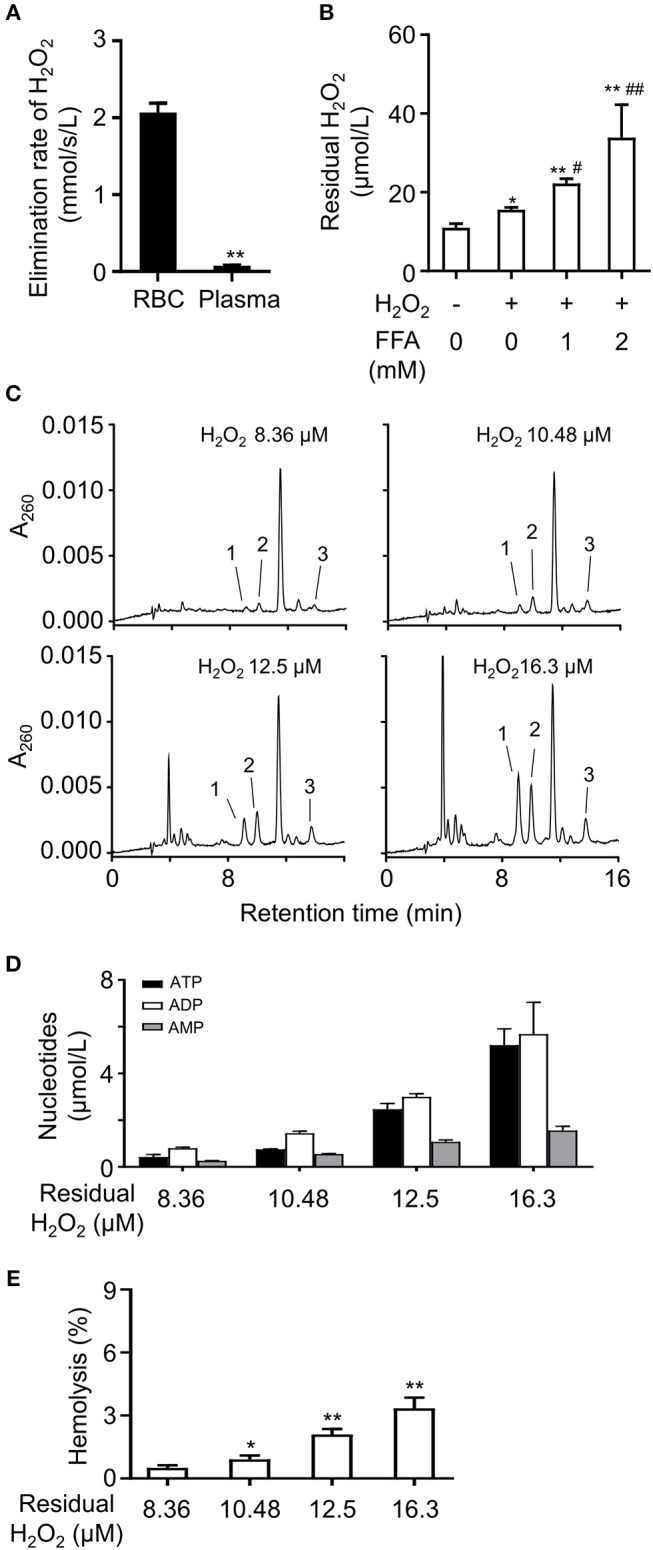
FFA induced the release of adenine nucleotides by lowering the elimination rate of H_2_O_2_ in RBCs. **(A)** Analysis of elimination rate of H_2_O_2_ in RBCs and plasma. **p < 0.01 compared with RBCs group (Student's t-test). **(B)** The mixture of FFA (OA: PA, 2:1) dose-dependently increased the level of residual H_2_O_2_ in RBCs. Representative HPLC profiles **(C)** and quantitation of ATP, ADP, and 5′-AMP **(D)** in breeding buffers of RBCs under various concentrations of residual H_2_O_2_. The release of nucleotides increased was relative to hemolysis induced by H_2_O_2_ **(E)**. Peak 1: ATP; peak 2: ADP, peak 3: 5′-AMP. Typical results of three independent experiments were plotted. *p < 0.05, **p < 0.01 compared to control group; ^#^*p* < 0.05, ^##^*p* < 0.01 compared with H_2_O_2_ group without FFA (one-way ANOVA with a Newman-Keuls *post hoc* test). All data were expressed mean ± S.D, *n* = 3 in each group.

It is well-known that FFA increase ROS production and elevate plasma ROS level (18, 19). RBCs play an important role in the defense against hydrogen peroxide (20). RBCs are under constant oxidative stress due to exposure to reactive oxygen species (ROS) produced inside and outside the cells. They contain non-enzymatic and enzymatic defense systems that scavenge ROS (21–23). To investigate the effects of FFA on the elimination rate of H_2_O_2_ in blood, RBCs and plasma were isolated and exposed to H_2_O_2_, respectively. The results showed RBCs had a significantly higher elimination rate of H_2_O_2_ compared with plasma (Figure 6A). Then we analyzed the influence of FFA on the elimination effect for H_2_O_2_. The addition of FFA decreased the elimination rate of H_2_O_2_ in RBCs, resulting in a higher level of residual H_2_O_2_ (Figure 6B). Next, we analyzed the effects of increasing H_2_O_2_ on nucleotides release from RBCs. RBCs were exposed to H_2_O_2_, then extracellular adenine nucleotides were analyzed by HPLC. The extracellular levels of ATP, ADP, and AMP were significantly increased with rising residual H_2_O_2_ (Figures 6C,D). This effect was relative to hemolysis (Figure 6E).

## Discussion

Several mechanisms have been proposed to explain how FFA inhibits insulin signaling. It is well-known that FFA activates the proinflammatory NF-κB pathway in skeletal muscle (24) and increases the expression of several cytokines including TNF-α, IL-1β, and IL-6 in liver (25), resulting in insulin resistance including activation of c-Jun NH2-terminal kinase (JNK) (26, 27). However, mice deficient in toll-like receptor 4 (TLR4) or TNF receptor with high-fat-diet still show significant insulin resistance (28, 29). Administration of TNF-α neutralizing antibody to patients with type 2 diabetes does not reduce their insulin resistance (30, 31), suggesting that the inflammatory process could be a result of obesity, but not a cause of insulin resistance or diabetes. In present studies, obese diabetic mice have higher level of plasma ANMs. Intragastric administration of olive oil induced glucose intolerance and elevation of ANMs in plasma. The dose of 5′-AMP affected the degree of glucose intolerance and blood glucose. Our results reveal plasma adenine nucleotides play an intermediary role on FFA-induced hyperglycemia in obese mice.

The rapid increase of plasma FFA can induce insulin resistance and then hyperglycemia (17). We have showed that FFA does not induce significant apoptosis under a short time. Thus, nucleotides release from cell damage caused by FFA may not be the sole source. Nucleotides are released from endothelial cells in the presence of OA in a short time, suggesting FFA also induces non-lytic release of nucleotides from endothelial cells. Moreover, glibenclamide partially inhibits ATP release induced by OA. Glibenclamide, an ATP-sensitive potassium channels inhibitor (32, 33), is widely used to improve insulin resistance and hyperglycemia in type 2 diabetes (34). Glibenclamide has been reported and used as an inhibitor of cystic fibrosis transmembrane conductance regulator (CFTR) and volume-regulated anion channels (VRAC) (33, 35–37). CFTR and VRAC are also considered as channels involved in ATP release (37–39) and expressed in HUVECs (40, 41). Thus, CFTR and VRAC act as potential channels involved in ATP release.

On the other hand, oxidative stress is increased in a diabetic state, and type 2 diabetes is associated with the accelerated production of ROS as well as a decreased scavenging of ROS (23, 42, 43). ROS, mainly H_2_O_2_, can inhibit insulin signaling (44). RBCs and vascular endothelial cells are the most universal cell types in circulation and in direct contact with high level of FFA in diabetes. Their contribution to adenine nucleotides release is more important than other cell types. Within RBCs, endogenous ROS are continuously generated by the autoxidation of hemoglobin (45). FFA strongly stimulate ROS production (46). RBCs also play a critical role in the defense against hydrogen peroxide (20). It has been reported that the activities of superoxide dismutase (SOD) and glutathione peroxidase (GSH-PX) involved in scavenging ROS are reduced in RBC after administration of fatty acid (47). Lowered activities of erythrocyte SOD and GSH-PX are also observed in type 2 diabetes (48). Thus, it is reasonable that FFA decreased RBCs elimination rate of H_2_O_2_. H_2_O_2_ could act as a stimulating factor in the release of adenine nucleotides from RBCs. Under a diabetic condition, erythrocytes are sensitive to oxidative stress, with increased susceptibility to hemolysis (49). Blood samples from *db/db* mice tend to exhibit hemolytic feature compared with those from wild type mice (50). Therefore, hemolysis induced by H_2_O_2_ is involved in the elevated extracellular adenine nucleotides.

In summary, our studies indicate that plasma adenine nucleotides play an intermediary role on FFA-induced hyperglycemia in obese mice.

## Data Availability

The raw data supporting the conclusions of this manuscript will be made available by the authors, without undue reservation, to any qualified researcher.

## Ethics Statement

This study was carried out in accordance with the recommendations of guidelines for the care and use of laboratory animals, Animal Care and Use Committee at Nanjing University of Science and Technology. The protocol was approved by Animal Care and Use Committee at Nanjing University of Science and Technology.

## Author Contributions

XY, YZ, and QS performed main research. YY, YG, WG, and JL analyzed data. XX wrote the part of paper. JZ designed the research and wrote the paper.

### Conflict of Interest Statement

The authors declare that the research was conducted in the absence of any commercial or financial relationships that could be construed as a potential conflict of interest.
